# Intravascular persistence of *Anaplasma platys, Ehrlichia chaffeensis,* and *Ehrlichia ewingii* DNA in the blood of a dog and two family members

**DOI:** 10.1186/1756-3305-7-298

**Published:** 2014-07-01

**Authors:** Edward B Breitschwerdt, Barbara C Hegarty, Barbara A Qurollo, Tais B Saito, Ricardo G Maggi, Lucas S Blanton, Donald H Bouyer

**Affiliations:** 1 Intracellular Pathogens Research Laboratory and the Center for Comparative Medicine and Translational Research, College of Veterinary Medicine, North Carolina State University, Raleigh, NC, USA; 2 University of Texas Medical Branch, Galveston, TX, USA

**Keywords:** *Anaplasma*, *Ehrlichia*, Rickettsemia, PCR, DNA sequencing

## Abstract

**Background:**

Anaplasmosis, caused by *Anaplasma phagocytophilum* and *Anaplasma platys,* and ehrlichiosis, caused by *Ehrlichia chaffeensis, Ehrlichia ewingii,* the "Panola Mountain *Ehrlichia*" and *Ehrlichia muris-*like pathogens have been identified as emerging tick borne infectious diseases in dogs and human patients. Persistent intravascular infection with these bacteria is well documented in dogs, but is less well documented in human beings.

**Methods:**

Serology and PCR targeting multiple microbial genes, followed by DNA sequencing, was used to test sequential blood samples. Tissue culture isolation was attempted in two laboratories.

**Results:**

*A. platys, E. chaffeensis,* and *E. ewingii* DNA was amplified from two *Anaplasma* and *Ehrlichia* seronegative family members and their dog, all lacking typical symptoms of anaplasmosis or ehrlichiosis. Following treatment with doxycycline, the dog and mother were *Anaplasma* and *Ehrlichia* spp. PCR negative.

**Conclusions:**

Sequential PCR testing provided molecular evidence supporting intravascular persistence of *A. platys* and *Ehrlichia* spp. in two humans and their dog. Diagnosticians and clinicians should consider the potential for co-infections due to these tick borne organisms.

## Background

Anaplasmosis, caused by *Anaplasma phagocytophilum* and *A. platys,* and ehrlichiosis, caused by *Ehrlichia canis*, *E. chaffeensis*, *E. ewingii, E. muris* and the “Panola Mountain *Ehrlichia*”*,* are emerging infectious diseases affecting dogs and human patients in North America and other parts of the world [1-3]. Canine vector borne diseases (CVBDs), including co-infections with more than one CVBD pathogen, are common in dogs, particularly those with frequent or constant vector exposure to varied tick species [4-6]*.* Among various animal species, including humans, *Anaplasma* and *Ehrlichia* spp. can induce acute, self-limiting or fatal infections, while persistent non-clinical infections often occur in dogs [7-9]*.* Following tick transmission, dogs can remain infected with *A. phagocytophilum, A. platys, E. canis, E. chaffeensis,* and *E. ewingii* for months to years, prior to immunological or therapeutic elimination of the infection or the development of chronic debilitating disease manifestations [7-9]*.* Dogs are natural hosts for *A. platys, E. canis* and potentially *E. ewingii,* but are considered opportunistic hosts for other *Anaplasma* and *Ehrlichia* spp. [6,7]*.* Persistent *E. chaffeensis*[10,11] and *E. canis*[12,13] human infections have been suspected previously. Recently, *A. platys* DNA was PCR amplified from blood samples collected from a veterinarian one month apart [14]. With the advent of PCR testing, reports of long-term *Anaplasma* and *Ehrlichia* human blood borne infections may become more common.

The data in this manuscript was generated after a physician requested to be entered into a *Bartonella* sp. research study. At time points spanning a six-month period, blood, buffy coat and inoculated cell cultures from members of the household, tested by PCR and sequencing, identified combinations of *A. platys* and *Ehrlichia* species. *Anaplasma platys*, *E. chaffeensis*, and *E. ewingii* DNA was amplified and sequenced from the dog, the physician and her daughter’s blood, but not from two other household members.

## Methods

### Patients and clinical presentation

In September 2011, a 57-year-old-female physician requested to be entered into an IRB approved research study (North Carolina State University, 164-08-05), investigating the prevalence of *Bartonella* sp. bacteremia in various patient populations. In 2008, the woman had developed intermittent subcutaneous edema and mildly increased liver enzyme activities. Her 16-year-old daughter, adopted from China at 6 months of age, had been healthy until 2008, after which she developed upper body muscle pain requiring treatment by a physical therapist. Between 2008 and 2011, both mother and daughter were examined by several specialist physicians; neither had symptoms or hematological abnormalities (thrombocytopenia) consistent with anaplasmosis or ehrlichiosis; and both were HIV negative.

In 2008 the family purchased a 15-week-old male Papillon from a Chicago pet store. The puppy originated from a Missouri breeding facility, a region endemic for tick borne *E. chaffeensis* and *E. ewingii*. Due to an acute illness, the puppy was hospitalized hours after purchase; pneumonia was diagnosed radiographically and an extended hospitalization period was required to effectively treat the pneumonia. Subsequently, aggressive behavior was observed, resulting in occasional bites of the mother and daughter but not the other household members. In 2012, the dog was diagnosed with microvascular dysplasia, a congenital, developmental abnormality common in small breed dogs, that can contribute to hepatic dysfunction and behavioral abnormalities, potentially explaining the tendency for the dog to bite.

### *Bartonella* alpha proteobacteria (BAPGM) enrichment blood culture/PCR

In accordance with a prior study [15]*,* three sample sets were collected within a 7-day period to enhance detection of *Bartonella* spp. DNA using the BAPGM platform. Using blood aseptically collected on Monday, Wednesday and Friday (mother in September 2011, December 2011 and January 2012), (daughter in January and February 2012) and (dog in December 2011 and January 2012), *Bartonella* spp. serology and BAPGM (*Bartonella* alpha Proteobacteria growth medium) enrichment blood culture testing for *Bartonella* spp. was performed, according to previously published protocols [15,16]*.*

### Buffy coat preparation

After the initial PCR amplification of *E. chaffeensis* DNA from the mother’s blood in September 2011, aseptically obtained ethylenediaminetetraacetic acid (EDTA)-anti-coagulated whole blood was processed in two formats (whole blood and buffy coat cells) in an effort to increase *Ehrlichia* sp. PCR sensitivity. Blood samples (mother, January, February and March) and (daughter, father and grandmother, March only) were sent concurrently to the NCSU-IPRL and UTMB for parallel tissue culture isolation attempts.

### *Anaplasma/ehrlichia* conventional PCR assays

Genomic DNA was extracted using either the QIAsymphony SP (Qiagen, Valencia, CA) or the DNeasy Blood & Tissue Kit (Qiagen, Valencia, CA) as per manufacturer recommendations. Each DNA extraction process included several negative control non-infected blood samples. To avoid DNA contamination of samples, DNA extraction and PCR sample preparation were performed in a room separate from the PCR amplification and gel analysis rooms, with a unidirectional work flow. Previously described *Anaplasma* and *Ehrlichia 16S* rRNA gene conventional PCR (cPCR) assays were used to test whole blood, buffy coat and cell culture supernatant fractions prepared from the mother, father, daughter, grandmother and dog [17,18]. Amplifications were performed in a Mastercycler EPgradient® aluminum block thermocycler (Eppendorf, North America). GEPs and GEPr and GEPs and GEP1060r primers were used respectively, to amplify 420 and 973bp segments of the *16S* rRNA gene [17] using *Ehrlichia canis* DNA as a positive control. Subsequently, newly introduced or derived (*sodb*) PCR assays were used by a blinded IPRL researcher to target the *A. platys p44* gene, *E. chaffeensis* and *E. ewingii p28* genes, and *Ehrlichia* spp. *sodb* gene in blood and cell culture supernatants. Primers and cPCR conditions are provided in Table 1. Amplicons were assessed by electrophoresis of 8 μl of each product in 2% agarose gels containing ethidium bromide. DNA extraction and PCR negative controls remained negative throughout the study.

**Table 1:** Conventional PCR primer sequences and thermocycler conditions for assays used in this study

**Target gene**	**Size in base pairs (bp)**	**Primers names and sequence, reaction mix and run conditions**
*Ehrlichia or Anaplasma***16S rRNA**	420 bp	**GEPs** 5’ CTG GCG GCA AGC YTA ACA CAT GCA AGT CGA ACG GA 3’ **GEPr** 5’ CTT CTT CTR TRG GTA CCG TCA TTA TCT TCC CYA YTG 3’
		For 25 μL mix: 12.5 μL MyTaq HS mix (Bioline), 0.2 μL 100 μM each primer (IDT® DNA Technology), 7.3 μl molecular grade water, + 5 μl template DNA. Denaturation 2 min @95°C, then 55 cycles 15 sec @94°C, annealing 10 sec @64°C, extension 15 sec @72°C, final extension 30 sec @72°C.
*Ehrlichia or Anaplasma***16S rRNA**	973 bp	**GEPs** 5’ CTG GCG GCA AGC YTA ACA CAT GCA AGT CGA ACG GA 3’ **LongGEP1060r** 5’CTG TGT RAG GTC CAG CCG AAC TGM SYC 3’
		As above except annealing and extension times extended to 25 and 30 sec
*Ehrlichia* spp. **sodb**	300 bp	**sodbF** 5’- TTT AAT AAT GCT GGT CAA GTA TGG AAT CAT **sodbR** 5’- AAG CGT GTT CCC ATA CAT CCA TAG
		For 50 μl mix: 24 μl MyTaq HS Mix (2X) (Bioline), 1 μl 50 uM each primer (Sigma-Aldrich), 4 μl molecular grade water + 20 μl template DNA. Single hot start cycle 3 min @95°C, then 55 cycles denaturation 10 sec @94°C, annealing 15 sec @58°C, extension 15 sec @72°C, then a single cycle 30 sec @72°C.
***E.chaffeensis*** **p28**	590 bp	**EchP28F** 5’- GAC CCA ACA GGT AGT GGT ATT AAC GG **EchP28R** 5’- CTG GGC TTA TAG AGT AGC TTA AAC CTA AC
		For 25 μL mix: 12.5 μl MyTaq HS Mix (2X) (Bioline), 0.25 μl 50 uM each primer (Sigma-Aldrich), 7 μl molecular grade water + 5 μl template DNA. Single hot start cycle 3 min @95°C, then 55 cycles denaturation 15 sec @94°C, annealing 15 sec @58°C, extension 30 sec @72°C, then a single cycle 30 sec @72°C.
***E. ewingii*** **p28**	215 bp	**EEM2F**^(Ref 32)^ 5′-GGA GCT AAA ATA GAA GAT AAT C **EEM1R** 5′-GTG CCA AAA GGT AAT ACA T
		For 25 μl mix: 12.5 μl of MyTaq HS Mix (2X) (Bioline), 0.25 μl 50 μM each primer (Sigma-Aldrich), 2 μl molecular grade water + 10 μl template DNA. Single hot start cycle 3 min @95°C, then 55 cycles denaturation at 15 sec @94°C, annealing 15 sec @56°C, extension 30 sec @72°C, then a single cycle 1 min @72°C.
***A. platys*** **p44**	520 bp	**Apl_p44F3** 5’- GCT AAG TGG AGC GGT GGC GAT GA CAG **Apl_p44R3** 5’- CGA TCT CCG CCG CTT TCG TAT TCT TC
		For 25 μL mix: 12.5 μl MyTaq HS Mix (2X) (Bioline), 0.3 μl 50 uM each primer (Sigma-Aldrich), 2 μl molecular grade water + 10 μl template DNA. Single hot start cycle 3 min @95°C, then 55 cycles denaturation 15 sec @94°C, annealing 10 sec @70°C, and extension 30 sec @72°C, then a single cycle 1 min @72°C.

### DNA sequencing

Amplicons were sequenced directly or cloned into plasmid pGEM-T Easy Vector System Promega® (Madison, WI) by GENEWIZ Inc. (Research Triangle Park, NC). Sequences were aligned and compared with GenBank sequences using AlignX software (Vector NTI Suite 6.0, InforMax, Inc.).

### Serologic testing

After amplification of *E. chaffeensis* DNA from the mother’s blood, her physician requested *E. chaffeensis* serology from a commercial laboratory (Quest Diagnostics, Nichols Institute, Valencia, CA); otherwise human serological testing was performed at the Rickettsial Disease Laboratory, UTMB. Serum specimens from all family members from multiple collection dates were serially diluted (twofold) from 1:64 to 1:2048 and tested for *E. chaffeensis* IgG class antibodies by ImmunoFluorescent assay (IFA) with Alexa Fluor 488 goat-anti-human secondary antibody (dilution 1:1000). An IFA titer of 1:64 or higher was considered positive. *E. chaffeensis* Western immunoblot was performed by SDS-PAGE using NUPAGE NoVEX 4-12% polyacrylamide gels (Invitrogen, Carlsbad, CA). Separated proteins transferred to nitrocellulose membrane using a Trans-Blot SD Semi-Dry Electrophoretic Transfer Cell (Bio-Rad Laboratories, Hercules, CA) were blocked overnight with 5% nonfat dry milk (Bio-Rad) in 1X Tris-buffered saline (TBS) before incubation with serum samples diluted (1:100) in 5% nonfat dry milk in 1X TBS overnight at room temperature. Blots were washed thoroughly then incubated with alkaline phosphatase labeled anti-human IgG (γ chain-specific) secondary antibodies and color reactions developed with BCIP/NBT phosphatase substrate (KPL, Inc. Gaithersburg, MD). Serum from the dog was tested for *Ehrlichia* antibodies by IFA in the IPRL, using antigens derived from canine monocytic DH82 cultures infected with *E. canis* and *E. chaffeensis* and for *Anaplasma* or *Ehrlichia* antibodies using the Snap® 4Dx®, (IDEXX Laboratories Inc., Westbrook, Maine) [19,20].

### Cell culture isolation

Parallel isolation attempts were made using white blood cell pellets (WBC) into various cell lines at NCSU-IPRL and UTMB. EDTA anti-coagulated whole blood from each individual was lysed with either ACK lysis buffer or hypotonic saline and centrifuged at UTMB or centrifuged in two stages at IPRL to prepare WBC pellets which were suspended in medium and dispersed into 25 cm^2^ flasks containing DH82 and IES6 tick cells (IPRL) or DH82, RF/6A, Vero, HEL299 and C6/36 cells (UTMB) and incubated at 37°C in 5% CO_2_. Negative control flasks were maintained in parallel. Cytology (Diff Quik or Gimenez staining) and PCR were sequentially performed.

## Results

### Exposure history

The family resided in suburban Chicago. There were no forested areas that might contain tick-infested deer for at least 1 mile in any direction. The mother did not recall any tick infestations in the home before or after purchasing the puppy and no family member reported a history of tick attachment. Occasional raccoon, skunk, opossum, coyote and other small mammals and birds were seen in the neighborhood. The mother and daughter were the primary caregivers for the dog and the only family members to have experienced consistent dog contact and occasional bites.

### Bartonella serology and enrichment culture/PCR

Based upon sequential testing, *Bartonella* spp. antibodies were not detected in the mother, daughter or dog’s serum specimens, and at all testing time points, BAPGM enrichment blood culture platform specimens (4 PCRs/sample/date of collection) were *Bartonella* sp. PCR negative. Thus, there was no serological or PCR evidence to support *Bartonella* spp. exposure or infection.

### *Anaplasma/Ehrlichia* conventional 16S rRNA PCR and sequencing

Using *16S* GEP primers that amplify both *Anaplasma* and *Ehrlichia* species, *Ehrlichia chaffeensis 16S* rDNA was cPCR amplified and sequenced from one of three September 2011 blood specimens (Table 2). The *16S* rDNA sequence shared 99.7% (359/360 base pair) similarity with *E. chaffeensis* strain Arkansas CP000236 (Table 3)*.* Based upon this *E. chaffeensis* result, additional blood samples from the mother, dog, daughter, father and maternal grandmother were submitted for testing in the IPRL on five, four, three, two and one occasion, respectively between September 2011 and March 2012.

**Table 2:** Summary of conventional PCR amplification and DNA sequencing results from whole blood, serum, WBC fractions and DH82, RF/6A or IES6 cell cultures tested between September 2011 and June 2012

**Date**	**Mother**			**Dog**				**Daughter**				**Father/Grandmother**			
	***Ech***	***Eew***	***Apl***	***Ech***	***Eew***	***Apl***	***Ec***	***Ech***	***Eew***	***Apl***	***Ec***	***Ech***	***Eew***	***Apl***	***Ec***
09/2011	+#	-	-	NT	NT	NT	NT	NT	NT	NT	NT	NT	NT	NT	NT
12/2011	+#	+*	+*	+#	-	-	-	NT	NT	NT	NT	NT	NT	NT	NT
01/2012	+#	+#	+#*	+#	-	+#*	-	-	+#	-	-	NT	NT	NT	NT
02/2012	+#	+#	-	-	+#	+*	-	-	-	+*	-	-	-	-	-
03/2012	+*	-	-	-	-	-	+*	+*	-	+*	-	-	-	-	-
06/2012**	-	-	-	-	-	-	-	-	-	-	-	NT	NT	NT	NT

Sample source or sources are designated for each testing time point.

*Ech* = *Ehrlichia chaffeensis, Eew* = *Ehrlichia ewingii, Apl* = *Anaplasma platys, Ec = Ehrlichia canis.*

All + results confirmed by DNA sequencing.

# = amplicon obtained from blood, serum or buffy coat fraction.

* = amplicon obtained from DH82, RF/6A or IES6 cell cultures.

NT = not tested.

** = post doxycycline treatment.

**Table 3:** **Confirmation of conventional PCR amplification results by DNA sequencing of *Ehrlichia *** **and *Anaplasma *** **gene targets derived from blood or WBC samples from the mother, dog and daughter**

**Gene target**^**a**^	**Mother**			**Daughter**			**Dog**		
	***Ech***	***Eew***	***Apl***	***Ech***	***Eew***	***Apl***	***Ech***	***Eew***	***Apl***
GEP *16S*	99.7	99.7	99.9	no	99.7	no	99.7	99.7	99.9
*sodB*	100	99.2	NA	no	100	NA	100	no	NA
*P28*	96.6	100	NA	no	100	NA	96.6	no	NA
*P44*	NA	NA	99.6	NA	NA	no	NA	NA	99.6

Based upon DNA sequence similarities, specimens from the dog and both family members contained the same genotypes.

*Ech* = *Ehrlichia chaffeensis.*

*Eew* = *E. ewingii.*

*Apl* = *Anaplasma platys.*

NA = not an applicable gene target for the particular *Anaplasma* or *Ehrlichia* species.

^a^Numbers represent % sequence identity to the following GenBank reference sequences: *A. platys 16S* M82801 from a dog, *A. platys p44* GQ868750.1 from a Venezuela dog strain, *E. chaffeensis 16S* CP000236, complete genome of an Arkansas human strain, *E. chaffeensis sodB* CP000236, *E. chaffeensis p28* human strains V8 (AF393394.1) and V4 (AF393390.1), *E. ewingii 16S* Accession NR_044747 from an Oklahoma dog strain, *E. ewingii sodB* (GenBank KC778986) from a naturally-infected dog procured through our diagnostic laboratory and validated by other *E. ewingii* gene targets, and *E. ewingii p28* AF287961.1 from a dog strain and AF287962.1, AF287963.1 and AF287966.1 from human strains.

Beginning with the mother’s December 2011 specimens, DNA was independently extracted from whole blood and WBC fractions from each family member in an effort to enhance cPCR sensitivity. By sequential testing, *E. chaffeensis* and *E. ewingii 16S* rDNA (99.7% similarity with NR044747) ehrlichiemia was documented in the dog, daughter and mother (Table 2). Also, at various time points, *A. platys 16S* rDNA (749/750 bp, 99.9% similarity to M82801) was amplified and sequenced from the dog and mother’s blood. Amplified *Anaplasma* and *Ehrlichia* DNA sequences shared between the dog, daughter and mother were identical.

### *Anaplasma/ehrlichia p28*, *p44* and *sodb* gene amplification and DNA sequencing

To confirm the *16S* rDNA PCR results, other *Anaplasma* and *Ehrlichia* spp. genes were targeted, using freshly extracted, stored, whole blood and WBC preparations. The initial *A. platys*, *E. chaffeensis* and *E. ewingii* DNA amplification results were confirmed by cPCR amplification and DNA sequencing of the *p44, p28,* and *sodb* genes (Table 3).

### Serology

When tested by a commercial laboratory in February 2012, the mother was reportedly *E. chaffeensis* seroreactive (IgM < 1:40, IgG 1: 160); however, at UTMB, five sera collected between September 2011 and March 2012 (mother) and February and March (daughter) were not *E. chaffeensis* seroreactive at a 1:64 dilution by IFA testing. Western immunoblotting also did not identify *Ehrlichia* spp. antibodies. When tested by the IPRL in January 2012, the dog was not *E. canis, Babesia canis, Babesia gibsoni, Bartonella henselae, Bartonella vinsonii* subsp. *berkhoffii, Leishmania infantum,* or *Rickettsia rickettsii* IFA seroreactive and was also ELISA negative for *Dirofilaria immitis* antigen and *Anaplasma* spp., *Borrelia burgdorferi* C6 peptide, and *Ehrlichia spp.* antibodies.

### Cell culture isolation

Organism isolation efforts at both NCSU and UTMB generated Diff Quik or Gimenez staining indications of intracellular bacteria or morulae, but did not result in successful isolation or evidence of organism amplification during 8–10 weeks in culture. Structures consistent with morulae were observed in the dog, daughter and mother’s inoculated cell cultures, but not in the father, grandmother or un-inoculated control cell cultures. *Anaplasma/Ehrlichia* GEP *16S* cPCR amplified *E. ewingii* and *A. platys* from the dog, daughter and mother’s IPRL DH82 cultures. *E. chaffeensis* DNA (16S *rRNA*, *sodb*, and *p28*) was amplified from daughter and mother’s UTMB RF/6A cultures. The dog and mother’s *E. chaffeensis* 16S *rRNA, sodb* sequences and *A. platys p44* sequences from cell culture were 100% identical to sequences previously amplifed from blood, serum or WBC specimens. The dog’s March 2012 WBC inoculated DH82 (pid 8) and IES6 (pid 23) cell cultures yielded 16S *rRNA* and *sodb E. canis* DNA.

### Treatment

In May 2012, the mother was treated with doxycycline 100 mg PO BID for 15 days and the dog was treated with doxycycline 5 mg/kg every 12 hours for 5 weeks. When retested in June 2012, blood and tissue culture cPCR results were negative and morulae-like structures were not visualized. The daughter was not treated.

## Discussion and conclusions

This study provided PCR amplification and sequencing evidence supporting the persistence of *A. platys, E. chaffeensis* and *E. ewingii* DNA in the blood of a dog and two family members. Specifically, during the six month study period, *E. chaffeensis*, *E. ewingii,* and *A. platys* DNA was amplified and sequenced from the mother’s blood and/or cell culture supernatant specimens at 5, 3, and 2 time points, respectively. *Anaplasma* and *Ehrlichia* gene targets not routinely assayed in our laboratory confirmed the initial identification of pathogen DNA in all three family members. Furthermore, the partial gene sequences amplified between family members were identical, suggesting they were infected with the same pathogen genotypes. Despite repeated molecular documentation of these bacteria, *Ehrlichia* spp. IFA antibody titers and Western immunobloting were negative at UTMB and the dog was not *Anaplasma* or *Ehrlichia* seroreactive at NCSU-IPRL. As *A. platys* and *E. ewingii* have not been successfully cultivated in cell culture, IFA serological assays were not available for testing purposes. Whether *Anaplasma* and *Ehrlichia* spp. co-infection altered the expected humoral immune response, whether assay antigens were not well matched with the infecting genotypes or whether anergy played a role in seronegativity remains unknown. Similar comparative correlations between canine and human vector-borne infections can be found in the literature. Persistent infections, spanning months to years, with *A. platys, E. chaffeensis* and *E. ewingii*, have been frequently reported in dogs [6,7,18,21]*.* In contrast, there is limited evidence supporting persistent human *E. chaffeensis* infections [10,11]. Based upon challenge studies, dogs can be experimentally re-infected with a homologous or heterologous *E. canis* isolate*,* thus infection does not infer protective immunity in dogs [22]. Re-infection with *E. chaffeensis* has also been reported in a liver transplant patient [23]. In conjunction with improved diagnostic testing modalities, co-infections of vector-borne diseases have been reported in dogs and in human patients. As dogs are more frequently exposed to ticks than their human counterparts, co-infections are more often reported in dogs [5-7,19] however, among other examples, *E. chaffeensis* and *Rickettsia rickettsii* co-infection was reported in a 44-year-old man [24]. Experimentally, *A. platys* and *E. canis* co-infections in dogs [18], influenced the patterns and severity of hematologic and serologic findings.

To confirm and validate the initial, unexpected *16S* rDNA PCR results from the mother and subsequently the daughter and dog, other gene targets were amplified and sequenced, isolation was attempted in two laboratories, and two investigators tested samples independently and at different time points. Because microbial-specific genes were targeted, the same PCR assays are applicable for testing human and veterinary patient populations and vectors for the presence of bacterial DNA. PCR has limitations. False negative results occur when testing samples with low template concentrations and selective amplification of the predominant organism can occur in patients co-infected with genetically similar organisms. By comparing PCR results from different sample sources (blood, serum, buffy coat, and tissue culture extracts), the assays used in this study may not have been sensitive enough to consistently detect these bacteria within blood specimens collected during the same week (data not shown), illustrating the need to enhance the sensitivity of *Anaplasma* and *Ehrlichia* PCR, particularly when patient samples contain DNA sequences of genetically related organisms. DNA carryover or amplicon contamination within a laboratory may result in false positives [25,26]. Importantly, throughout this study, all DNA extraction and PCR-negative controls remained negative. Furthermore, PCR testing of blood, serum, WBC, and tissue culture extracts from the father or maternal grandmother did not identify *Anaplasma* and *Ehrlichia* sp. DNA, and both the dog and mother became PCR negative after treatment with doxycycline, a finding that supports infection with viable organisms.

Nearly two decades ago investigators in Venezuela described inclusions in human platelets, a subset of which were ultrastructurally consistent with *A. platys*[27,28]. Until recently [14], no subsequent report of human *A. platys* infection was published in the English literature. There is substantial epidemiological support for *Rhipicephalus sanguineus* (the brown dog or kennel tick) as the vector and the dog as the primary reservoir host for *A. platys* and *E. canis*[20]. As the puppy in this study originated from a kennel in Missouri, exposure to *R. sanguineus* seemed more likely than exposure to *A. americanum,* which is a field tick that will feed on numerous animal species [29]. Although vector competence has not been proven, *E. chaffeensis* DNA was amplified from 56% of *R. sanguineus* obtained from dogs and puppies housed in a kennel in Cameroon, a country where both canine and human infection with *E. chaffeensis* has been previously reported [30]. In contrast, recent efforts to experimentally transmit *E. ewingii* by *R. sanguineus* were not successful [31]. The source of *A. platys, E. chaffeensis* and *E. ewingii* infection in the pet and the family members and *E. canis* in the dog remains unknown; however, this puppy originated from a kennel in a highly endemic state for tick transmission of *E. chaffeensis* and *E. ewingii* by *A. americanum*[1,29] and *A. platys* and *E. canis* are transmitted by a tick (*R. sanguineus*) that infests dogs in kennels*.*

In summary, *A. platys* and *Ehrlichia* spp. DNA was documented in an unusual familial cluster involving a dog and two family members. Future studies should determine whether repeated documentation of DNA of these organisms reflects ongoing infection and whether there are medical consequences associated with the persistence of DNA of these organisms. To guide testing and treatment decisions, sequential PCR testing of blood, WBC or cell culture-enhanced samples may increase DNA-detection sensitivity.

## Abbreviations

BAPGM: Bartonella alpha proteobacteria growth medium; EDTA: Ethylenediaminetetraacetic acid.

## Competing interests

In conjunction with Dr. Sushama Sontakke and North Carolina State University, Dr. Breitschwerdt holds U.S. Patent No. 7,115,385; Media and Methods for cultivation of microorganisms, which was issued October 3, 2006. He is the chief scientific officer for Galaxy Diagnostics, a company that provides advanced diagnostic testing for the detection of *Bartonella* species infection in animals and humans. All other authors have no potential conflict. In the context of this manuscript, none of the authors disclose any conflicts of interest.

## Authors’ contribution

EBB coordinated communications and generated the initial draft manuscript. BCH, BAQ, and RGM performed tissue culture isolation and PCR testing at North Carolina State University. TBS, LSB, and DHB performed tissue culture isolation and serological testing at the University of Texas Medical Branch at Galveston. All authors contributed to the content of the manuscript and all authors reviewed the final submission.
